# Ketamine and Active Ketamine Metabolites Regulate STAT3 and the Type I Interferon Pathway in Human Microglia: Molecular Mechanisms Linked to the Antidepressant Effects of Ketamine

**DOI:** 10.3389/fphar.2019.01302

**Published:** 2019-11-05

**Authors:** Ming-Fen Ho, Cheng Zhang, Lingxin Zhang, Hu Li, Richard M. Weinshilboum

**Affiliations:** Division of Clinical Pharmacology, Department of Molecular Pharmacology and Experimental Therapeutics, Mayo Clinic, Rochester, MN, United States

**Keywords:** ketamine, gene expression, microglia, RNA-seq, antidepressant

## Abstract

Inflammation is an important biological process which contributes to risk for depression, in part as a result of the production of proinflammatory cytokines and of alterations in glutamatergic neurotransmission. Ketamine has anti-inflammatory properties which might contribute to its antidepressant effects. This study was designed to clarify mechanisms of action for ketamine and its active metabolites, (2*R,6R;2S,6S*)-hydroxynorketamine (HNK), which also appear to play a major role in ketamine’s rapid antidepressant effects. An HMC3 human microglial cell line was used as a model system to test a possible role for ketamine in immune response regulation that might contribute to its antidepressant effects. Our results highlight the fact that ketamine and its two active metabolites can regulate the type I interferon pathway mediated, at least partially, through signal transducer and activation of transcription 3 (STAT3) which plays a major role in the immune response. Specifically, STAT3 downstream genes that were modulated by either ketamine or its active metabolites were enriched in the “response to type I interferon” pathway. Our data also suggest that STAT3 might play a role in ketamine’s antidepressant effects, mediated, at least in part, through eukaryotic elongation factor 2 (EEF2), resulting in the augmentation of brain-derived neurotropic factor (BDNF) expression and promoting the synthesis of synaptic proteins postsynaptic density protein 95 (PSD95) and synapsin I (SYN1).

## Introduction

Neuroinflammation contributes significantly to the pathogenesis of depression, in part through the activation of microglia, cells that play a key role in the immune response in the brain (Dantzer et al., 2008). Specifically, patients with treatment resistant depression display elevated levels of inflammatory cytokines and chemokines such as interleukin 6 (IL6) and monocyte chemoattractant protein 1 (MCP-1) (Kiraly et al., 2017). It has also been reported that the antidepressant drug ketamine has anti-inflammatory effects (Kiraly et al., 2017), but the underlying molecular mechanism is not well understood. Previous studies have shown that ketamine-induced antidepressant effects involve the activation of multiple signaling pathways including the mTOR/AKT and ERK pathways (Mion and Villevieille, 2013; Sleigh et al., 2014). Activation of mTOR signaling is one of the most intensively studied of the processes that might contribute to the antidepressant effects of ketamine (Li et al., 2010; Harraz et al., 2016; Yang et al., 2018). Specifically, ketamine blocks N-methyl-D-aspartate (NMDA) receptors, and its active metabolites [*2R,6R*-HNK (hydroxynorketamine) and *2S,6S*-HNK] also contribute to its antidepressant effects, at least in part through the activation of α-amino-3-hydroxy-5-methyl-4-isoxazolepropionic acid (AMPA) receptors (Collo et al., 2018; Collo et al., 2019). It has also been shown that ketamine infusion results in increased plasma glutamate concentrations in patients with refractory major depressive disorder (MDD) (Rotroff et al., 2016). Glutamate, in turn, can bind to AMPA receptors to induce depolarization as well as sodium and calcium influx through a process that involves AMPA receptors and L-type voltage gated calcium channels. This series of events results in increased brain-derived neurotropic factor (BDNF) release from synaptic vesicles. BDNF binds with high affinity to tyrosine kinase receptor B (TrkB) which leads to activation of both the ERK and AKT/mTOR pathways, increasing protein translation of postsynaptic density protein 95 (PSD95) and synapsin I (SYN1). This, in turn, facilitates synaptogenesis, and produces antidepressant and anxiolytic behavioral effects (Liu et al., 2013; Sarkar and Kabbaj, 2016).

In addition to these mechanisms, several preclinical studies have reported that female experimental animals responded to ketamine treatment better than did males (Carrier and Kabbaj, 2013; Franceschelli et al., 2015; Zanos et al., 2016; Sarkar and Kabbaj, 2016). We recently reported that ketamine and its two active metabolites are ligands for estrogen receptor alpha (ERα) (Ho et al., 2018), which might contribute to sex-related differences in ketamine efficacy (Carrier and Kabbaj, 2013; Franceschelli et al., 2015; Sarkar and Kabbaj, 2016; Zanos et al., 2016). Two thirds of MDD patients are women, and sex hormones themselves are considered inflammatory mediators, i.e., estrogens can enhance humoral immunity (Marques-Deak, et al., 2005). The role of estrogen in neuropsychiatric disorders is complex because estrogens have both neuroprotective and neuroexcitatory actions and they have been recognized as a factor that can influence both neurotransmission and immunity (Gillies and McArthur, 2010). In summary, the antidepressant effects of ketamine might be mediated, as least in part, as a result of the anti-inflammatory effects of ketamine with decreased production of proinflammatory cytokines. Against this background, we hypothesized that ketamine might influence the expression of inflammatory mediators and pathways of inflammation in microglia—cells that play a major role in immune response in the brain.

The present study was designed to 1) identify molecular signatures for ketamine and its two active metabolites in human microglial cells, the predominant immune cells in the brain which play a central role in immune response regulation in the central nervous system, and 2) to determine possible molecular mechanisms underlying ketamine’s antidepressant effects. Our studies began with a determination of genome-wide expression profiles of HMC3 cells in response to treatment with either ketamine or the two known ketamine active metabolites (2*R*,6*R*)-HNK or (2*S*,6*S*)-HNK—alone or in combination with estradiol (E2). All drug treatment conditions were optimized as described in our previous study (Ho et al., 2018). Pathway analysis of those genome-wide expression data placed a focus on the type I interferon pathway and, as a result, on the immune response. Included among the upregulated genes was signal transducer and activation of transcription 3 (STAT3), a transcription factor known to be involved in regulation of the interferon pathway (Ivashkiv and Donlin, 2014). The STAT3 DNA binding motif sequence was enriched in the genes that we found to be regulated by ketamine and its active metabolites. We also observed that the induction of STAT3 by ketamine resulted in STAT3 nuclear translocation and associated alterations in transcription regulation for a series of immune mediators involved in the “response to type I interferon” pathway. All of these observations were compatible with our results after the treatment of HMC3 cells with ketamine and its two active metabolites. We then pursued underlying mechanism(s) by which STAT3 nuclear translocation after ketamine treatment might have resulted in altered transcriptional regulation of immune mediators in the “response to type I interferon” pathway. We also demonstrated that STAT3 interacted with eukaryotic elongation factor 2 (EEF2) which can be regulated by EEF2 kinase, another protein known to play a role in the rapid antidepressant effects of ketamine (Monteggia et al., 2013). All of these observations served to emphasize the possible importance of STAT3 in the antidepressant effects of ketamine. As a result, this series of studies, as described subsequently, have enhanced our understanding of ketamine’s mechanism of action, with a focus on immune response in microglia—cells that play a major role in immunity in the brain.

## Materials and Methods

### Cell Culture and Drug Treatment

HMC3 cells (ATCC CRL-3304, Manassas, VA, USA) were cultured in Eagle’s Minimum Essential Medium (EMEM) (Cellgro, Manassas, VA, USA) supplemented with 10% fetal bovine serum (FBS) (Atlanta Biologicals, Flowery Branch, GA, USA). Ketamine hydrochloride was purchased from Pfizer (New York, NY, USA). Both (*2R,6R*)-2-amino-2-(2-chlorophenyl)-6-hydroxycyclohexanone hydrochloride (*2R,6R*-HNK) (lot number: NCGC00378227-18), and (*2S,6S*)-2-amino-2-(2-chlorophenyl)-6-hydroxycyclohexanone hydrochloride (*2S,6S*-HNK) (lot number: NCGC00373033-12) were synthesized and characterized in Dr. Craig Thomas’s laboratory at the National Center for Advancing Translational Sciences (Rockville, MD, USA). Before treatment with ketamine or the two active metabolites, cells were cultured in EMEM media containing 5% (V/V) charcoal stripped FBS for 24 h, and were subsequently cultured in FBS free Roswell Park Memorial Institute (RPMI) media for another 24 h. Cells were then treated with ketamine or with the ketamine metabolites (2*R*,6*R)*-HNK or (2*S*,6*S)-HNK* for 24 h in serum and phenol red-free medium. In some experiments, cells were exposed to 0.1 nM E2 (Sigma, St. Louis, MO, USA) together with 400 nM ketamine, (*2R,6R*)-HNK, or (*2S,6S*)-HNK, at concentrations similar to plasma concentrations observed during ketamine therapy (Zarate et al., 2012).

### RNA Sequencing and Data Analysis

Total RNA was extracted using the RNeasy mini kit (Qiagen, Valencia, CA, USA). RNA-seq experiments were conducted by the Mayo Clinic Center for Individualized Medicine Medical Genomics Facility. RNA-seq libraries were prepared with Ovation RNA-seq system v2 kit (NuGEN) according to the manufacturer’s instruction, and were sequenced using an Illumina HiSeq 2000 with eight samples in each lane using 100 bp paired end index reads. Fastq files containing paired RNASeq reads were aligned with Tophat 2.0.12 (Kim et al., 2013) against the University of California Santa Cruz (UCSC) human reference genome (hg19) using Bowtie 2.2.3 with default settings (Langmead and Salzberg, 2012). Gene level counts from uniquely mapped, non-discordant read pairs were obtained using the subRead featureCounts program (v1.4.6) (Liao et al., 2013) and gene models from the UCSC hg19 Illumina iGenomes annotation package. Differential expression analysis was also performed using the DESeq2 package with default parameters (Love et al., 2014). Pathway analysis was performed using gene set enrichment analysis (GSEA) software (Mootha et al., 2003; Subramanian et al., 2005). Gene ontology (GO) terms for each dataset were generated using The Database for Annotation, Visualization and Integrated Discovery (DAVID) v6.8 (https://david.ncifcrf.gov/) (Huang et al., 2008). RNA-sequencing data are available *via* the GEO accession number: GSE134782.

### Real Time PCR

The PCR reactions contained 100 ng of total RNA, 5 µl of 2X VeriQuest SYBR green qPCR master mix (Affymetrix, Santa Clara, CA USA), 0.1 µl of DNA polymerase, 1 µl of gene specific primer, and distilled water up to 10 µl final volume per reaction. Primer sets for real-time PCR are listed in **Supplementary Table 1**. Real time PCR reactions were performed in duplicate using the Applied Biosystems ViiA *7*™ Real-Time PCR System (Life Technologies, Carlsbad, CA, USA). The 2^-ΔΔCt^ method was employed for statistical data analysis.

### Immunofluorescence Staining and Confocal Imaging Analysis

HMC3 cells were grown on glass coverslips and treated with ketamine or the two active metabolites for 24 h. Cells were then fixed in 4% paraformaldehyde at room temperature for 15 min. Cells were washed in cold phosphate-buffered saline (PBS) and permeablized with 0.2% Triton X-100 in PBS. After blocking for 1 h with 3% bovine serum albumin (BSA), cells were incubated with anti-STAT3 or anti-SIN3A antibodies overnight at 4°C. The secondary antibody was used at 1:1,000 dilution for an hour. Antibodies for immunofluoresence are listed in **Supplementary Table 1**. 4′,6-diamidino-2-phenylindole (DAPI) at a concentration of 1.43 µM was used to stain the cell nuclei (blue). Slides were visualized using fluorescence microscopy (Olympus, FV1200).

### Immunoprecipitation, Mass Spectrometry, and Western Blot Analysis

HMC3 cells (1 × 10^7^) were resuspended in 1 ml immunoprecipitation (IP) lysis buffer containing 5 µl protease inhibitor cocktail (Qiagen, Valencia, CA, USA) and were incubated on ice for 30 min. Cells were then centrifuged at 12,000 *g* at 4°C for 15 min. Supernatants were collected. Protein A agarose (ThermoScientific, Madison, WI, USA) was prepared and washed with IP lysis buffer. A pre-cleaning step was performed in order to clean the background. Cell lysates containing protein A agarose beads were rotated at 4°C for an hour. Supernatant was collected after centrifugation. At this point, input (50 µl) was collected and stored at −80°C. Anti-STAT3 (1:50) antibody (Cell Signaling Technology, Danvers, MA, USA) was used to perform IP. IgG (abcam, Cambridge, MA, USA) was used as negative control. Specifically, IP samples containing protein A agarose beads were rotated at 4°C overnight. Immunoprecipitates were washed three times with ice cold lysis buffer, and proteins were eluted with 50 µl 2X Laemmli loading buffer. Proteins pulled down by anti-STAT3 antibody were separated on 4–12% sodium dodecyl sulfate polyacrylamide gel electrophoresis (SDS-PAGE) gels for mass spectrometry (The Taplin Biological Mass Spectrometry Facility, Boston, MA, USA). In some experiments, proteins were transferred onto polyvinylidene difluoride (PVDF) membranes. After blocking, membranes were incubated with primary antibodies against STAT3 or SIN3A or EEF2 at 4°C overnight. The washed membranes were then incubated with secondary antibody (1:30,000 dilution) for an hour at room temperature. The membranes were visualized using super signal ECL substrate (Thermo Scientific, Madison, WI, USA). In some experiments, cytoplasmic and nuclear extracts were isolated using nuclear extract kit following the manufacture’s protocol (Active Motif, Carlsbad, CA, USA).

### STAT3 Chromatin Immunoprecipitation (ChIP) Assays

ChIP assays were performed using HMC cells before and after drug treatment using the EpiTect ChIP OneDay Kit (Qiagen, Valencia, CA, USA). DNA-STAT3 complexes were immunoprecipitated using antibodies against STAT3 or with normal mouse IgG as a control. Real-time PCR was used to quantify STAT3 binding. Primer sets and antibodies for ChIP assays are listed in **Supplementary Table 1**. The level of enrichment was expressed as relative enrichment above background (enrichment relative to IgG control). Data were represented as % input.

### Statistics

Real-time PCR results were analyzed using ANOVA, followed by Tukey’s multiple comparison tests for individual comparisons when significant effects were detected. Gene expression data were presented as mean ± SEM. Differences were considered significant at *p* < 0.05. GraphPad Prism Software v7 (San Diego, CA, USA) was used for data analysis.

## Results

### Gene Expression Profiles in Microglia Treated With Ketamine or Its Active Metabolites

As a first step in the present study, we set out to characterize molecular signatures of exposure to ketamine and its two active metabolites. Specifically, our study was designed to determine—as a first step—genome-wide mRNA expression profiles for HMC3 cells in response to treatment with ketamine and its two active metabolites with and without exposure to E2. Drug treatment conditions had been optimized as described in our previous study (Ho et al., 2018). Specifically, the concentrations of E2 (0.1 nM), ketamine and ketamine metabolites (400 nM) used to perform these experiments were selected to fall within the physiological range for E2, and within the range of concentrations of ketamine and ketamine metabolites observed during ketamine infusion therapy for patients suffering from depression (Zarate et al., 2012; Zhao et al., 2012).

**Figure 1:**
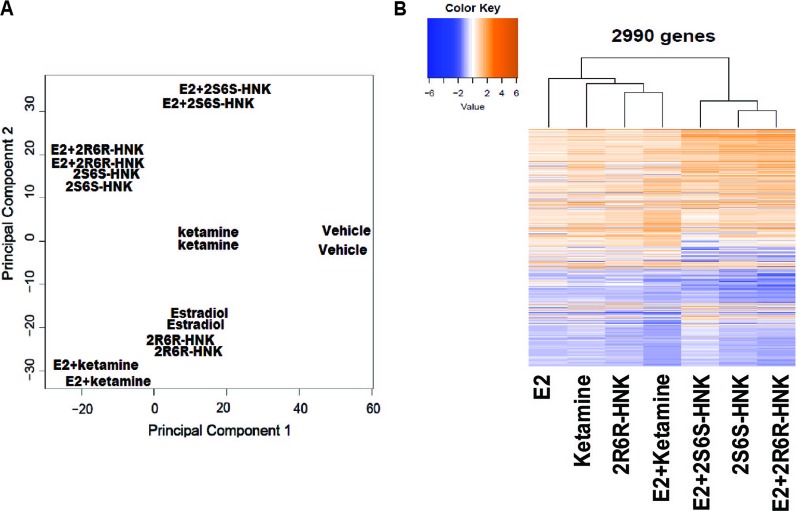
**(A)** Principal components analysis (PCA) of gene expression profiles in HMC3 cells after exposure to vehicle, estradiol (E2), ketamine, (*2R,6R*)-HNK, (*2S,6S*)-HNK, E2+ketamine, E2+(*2R,6R*)-HNK, and E2+(*2S,6S*)-HNK. **(B)** Heatmap showing expression profiles for 2,990 selected genes regulated by E2, ketamine or its metabolites.

Principal component analysis (PCA) of gene expression profiles showed distinct clustering for the differing drug treatment conditions, i.e. in the presence of E2 alone or together with either ketamine or the ketamine metabolites (**Figure 1A**). Heat map plots showed 2,990 genes for which expression was significantly altered [log2 fold change ≥1 or ≤−1, false discovery rate (FDR) < 0.05] after drug treatment as compared to vehicle treatment (**Figure 1B**). We then performed pathway analysis for each drug treatment condition. Strikingly, “response to type I interferon pathway” was the most common and most highly affected pathway in the presence of ketamine or its two active metabolites, with or without E2, all of which differed from E2 treatment alone (**Table 1**).

**Table 1:** Pathway analysis.

Treatment	Pathway	Size	p value	FDR
**E2**	Terpenoid metabolic process	58	<1.0E−08	0.002272
	Synaptic transmission cholinergic	15	<1.0E−08	0.003637
	Negative regulation of coagulation	33	<1.0E−08	0.005619
	Calcium-independent cell-cell adhesion via plasma membrane cell-adhesion molecules	14	<1.0E−08	0.005923
	Regulation of calcium ion-dependent exocytosis	54	<1.0E−08	0.008802
**Ketamine**	Response to type I interferon	48	<1.0E−08	<1.0E−08
	Terpenoid metabolic process	58	<1.0E−08	8.26E−05
	Synaptic signaling	251	<1.0E−08	6.2E−05
	Humoral immune response	61	<1.0E−08	0.000104
	Cell-cell signaling	432	<1.0E−08	0.000379
**(2R,6R)-HNK**	Response to type I interferon	48	<1.0E−08	<1.0E−08
	Negative regulation of viral genome replication	39	<1.0E−08	0.002157
	Negative regulation of coagulation	33	<1.0E−08	0.002622
	Responses to interferon-gamma	83	<1.0E−08	0.005928
	Unsaturated fatty acid biosynthetic process	37	<1.0E−08	0.006488
**(2S,6S)-HNK**	Response to type I interferon	48	<1.0E−08	<1.0E−08
	Synaptic signaling	251	<1.0E−08	0.00163
	Neurotransmitter transport	95	<1.0E−08	0.001959
	Neuropeptide signaling pathway	32	<1.0E−08	0.001951
	Presynaptic process involved in synaptic transmission	79	<1.0E−08	0.002952
**E2+ketamine**	Response to type I interferon	48	<1.0E−08	<1.0E−08
	Icosanoid metabolic process	53	<1.0E−08	0.000159
	Fatty acid derivative metabolic process	53	<1.0E−08	0.000387
	Responses to interferon-gamma	83	<1.0E−08	0.000364
	Neuropeptide signaling pathway	32	<1.0E−08	0.001181
**E2+(2R,6R)-HNK**	Neuropeptide signaling pathway	32	<1.0E−08	0.002072
	Calcium-independent cell-cell adhesion via plasma membrane cell-adhesion molecules	14	<1.0E−08	0.00527
	Regulation of granulocyte chemotaxis	26	0.001815	0.009032
	Response to type I interferon	48	<1.0E−08	0.009505
	Potassium ion transport	84	<1.0E−08	0.011445
**E2+(2S,6S)-HNK**	Response to type I interferon	48	<1.0E−08	0.045259
	Citrulline metabolic process	9	0.001869	0.043259
	Neuropeptide signaling pathway	32	<1.0E−08	0.042126
	Muscle filament sliding	25	0.001658	0.044351
	Positive regulation of neutrophil migration	19	0.001656	0.044662

### Ketamine Influenced the Type I Interferon Pathway in Human Mmicroglia

**Figure 2:**
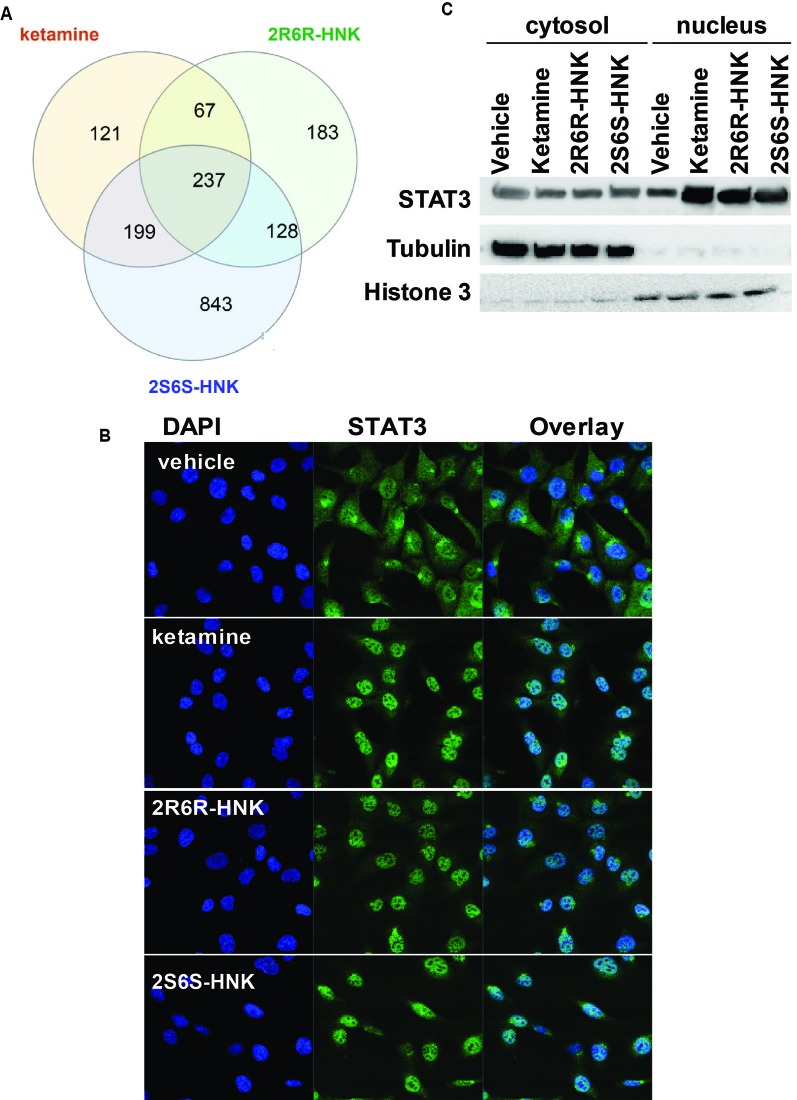
**(A)** Venn diagram showing the number of genes in HMC3 cells which were significantly altered by exposure to ketamine or the two active ketamine metabolites (*2R,6R*)-HNK and (*2S,6S*)-HNK as determined by RNA-seq. The STAT3 DNA binding motif sequence was enriched in the genes that we found to be regulated by ketamine and its active metabolites. **(B)** Immunofluorescence staining of HMCs cells showing the nuclear translocation of STAT3 after the cells were treated with ketamine (400 nM) or its two active metabolites (400 nM). **(C)** Western blot analyses of cytosolic and nuclear fractions derived from HMC3 cells before and after drug exposure. Three biological replicates were performed.

**Figure 3:**
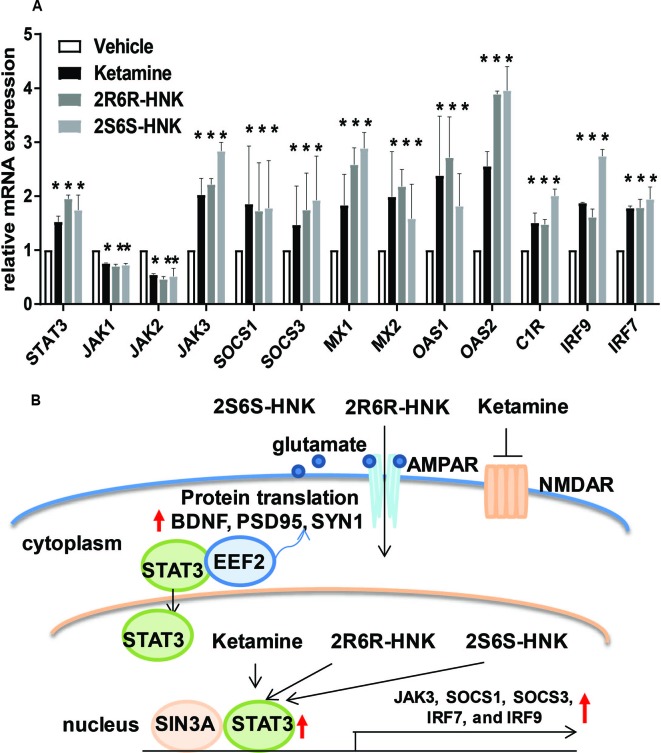
**(A)** mRNA expression of genes involved in the interferon alpha and beta signaling pathways in HMC3 cells before or after exposure to ketamine or the two active ketamine metabolites (*2R,6R*)-HNK or (*2S,6S*)-HNK. ANOVA was performed to compare gene expression, followed by Tukey’s multiple comparisons correction for significant effects. *p ≤ 0.05, as compared to vehicle treatment. Results are represented as mean ± SEM of three independent determinations. **(B)** Schematic diagram illustrating the effects of STAT3 in response to exposure to ketamine or the two active ketamine metabolites in HMC3 cells. Specifically, STAT3 translocation can occur in the presence of ketamine or its two active metabolites which, in turn, alters the expression of downstream genes in the type I interferon pathway. These STAT3 effects appeared to be mediated, at least partially, through EEF2, a protein known to regulate BDNF, PSD95, and SYN1 protein translation, all of which have been reported to contribute to ketamine’s antidepressant effects.

We next identified 237 genes that were significantly regulated by both ketamine and the two active ketamine metabolites, (2*R*,6*R*)-HNK and (2*S*,6*S*)-HNK (**Figure 2A**). We then performed pathway analysis for these 237 genes (**Supplementary Table 2**). Once again, strikingly, the same signaling pathway (response to type I interferon) was observed with a *p* value of 4.8E−3, and even more striking, a DNA motif sequence “TTCNNNGAA” for the STAT family was found to be significantly enriched in the promoter regions of those 237 genes with an FDR < 0.05 as determined by GSEA software (Mootha et al., 2003; Subramanian et al., 2005). It is known that the STAT family of proteins plays a major role in the immune response and that these proteins are important mediators of interferon signaling (Chen et al., 2004; Au-Yeung et al., 2013; Ivashkiv and Donlin, 2014). Therefore, we hypothesized that the STAT family might be involved in transcription regulation during ketamine treatment, so we set out to determine the subcellular localization of members of the STAT family before and after ketamine exposure. Strikingly, in the presence of ketamine, STAT3 was translocated into the nucleus where it could act as a transcription factor (**Figure 2B**). However, STAT1, STAT2, STAT5A, STAT5B, and STAT6 did not alter their subcellular localization in response to ketamine treatment (data not shown). STAT4 is not expressed in HMC3 cells. STAT3 nuclear translocation was also observed in the presence of (2*R*,6*R*)-HNK or (2*S*,6*S*)-HNK in the same cell line (**Figures 2B, C**). Furthermore, STAT3 mRNA nuclear expression was induced by exposure to ketamine and (2*R*,6*R*)-HNK or (2*S*,6*S*)-HNK in human microglial HMC3 cells (**Figure 3A**). We used the same set of samples to verify the expression of a series of genes identified during our RNA-seq studies, genes that were involved in the type I interferon signaling pathway (**Figure 3A**). The expression of all of these genes was significantly regulated by ketamine and (2*R*,6*R*)-HNK or (2*S*,6*S*)-HNK.

Since STAT3 binding motif sequences were enriched in the promoter regions of genes that significantly changed their expression in response to exposure to ketamine and its two active metabolites (**Figure 2A**), we hypothesized that the ketamine-dependent alteration in transcription for genes involved in the type I interferon signaling pathway might be mediated, at least in part, through the induction of STAT3 binding. If so, we set out to determine and characterize whether any STAT3-interacting partner proteins might also be involved in “ketamine-mediated gene expression regulation” which might be associated with the antidepressant effects of ketamine as illustrated in the working model depicted schematically in **Figure 3B**. To test that possibility, we next performed siRNA knockdown studies before and after exposure to ketamine and its two active metabolites.

### STAT3 Influenced the Type I Interferon Pathway During Ketamine Treatment

**Figure 4:**
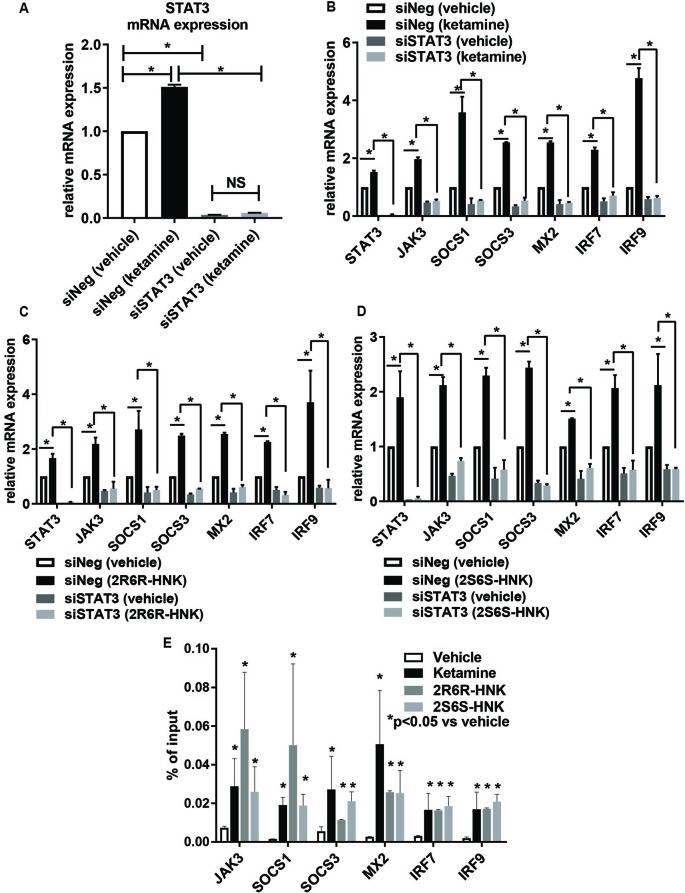
**(A)** Knockdown of STAT3 using siRNA resulted in the downregulation of STAT3 and failure to display STAT3 induction by ketamine. The induction of gene expression by ketamine **(B)** or the two active ketamine metabolites (*2R,6R*)-HNK **(C)** or (*2S,6S*)-HNK **(D)** for a series of genes involved in the interferon alpha and beta signaling pathways was abolished after STAT3 was knocked down. *p ≤ 0.05. Three biological replicates were performed. **(E)** ChIP assays showing the effect of STAT3 binding to the DNA binding motif. The level of enrichment is expressed as relative enrichment above background (enrichment relative to IgG control). Data are represented as % input, (enrichment relative to IgG control) = % input (STAT3 antibody) − % input (IgG). *p ≤ 0.05 vs. vehicle. Results are represented as mean ± SEM of three biological replicates.

To make it possible to study the possible role of STAT3 in the type I interferon pathway, siRNA knockdown studies were performed using four independent siRNAs as well as one pooled siRNA (Dharmacon Chicago, IL, USA). The results for all of those experiments were consistent. When STAT3 was knocked down to ∼3% of baseline levels, ketamine failed to activate the expression of STAT3 (**Figure 4A**). In parallel, in the presence of ketamine or its active metabolites, the pattern of induction of gene expression for type I interferon signaling target genes was also abolished or dramatically decreased after STAT3 knockdown (**Figures 4B–D**). Specifically, silencing STAT3 by siRNA resulted in the down-regulation of the expression of JAK3, SOCS1, SOCS3, MX2, IRF7, and IRF9, as compared to control siRNA (**Figure 4B**). Furthermore, induction of the same set of genes by either ketamine or the active ketamine metabolites was lost when STAT3 was silenced by siRNA treatment (**Figures 4B–D**). These results are compatible with the conclusion that ketamine and its active metabolites can induce the expression of STAT3 and, as a result, they can activate genes encoding proteins in the type I interferon pathway.

We next set out to determine whether ketamine could alter STAT3 binding, thus influencing downstream transcription regulation (**Figures 4B–D**) for genes which contained STAT3 binding sites within their promoter regions as previously reported based on genome-wide STAT3 ChIP-seq studies (Tripathi et al., 2017; Davis et al., 2018). We performed STAT3 ChIP assays for six of these genes and confirmed that STAT3 occupancy increased significantly in the presence of ketamine (**Figure 4E**), results compatible with the gene expression patterns shown in **Figure 4B**. Similar results were observed in the presence of the two active ketamine metabolites (**Figures 4C–E**). STAT3 is a transcription factor, as supported by the data shown graphically in **Figure 4E**, but whether that is the only molecular mechanism involved in its relationship to ketamine’s effects was unclear. Therefore, the next series of experiments was designed to test the possibility that protein-protein interactions which might also contribute to “ketamine-mediated transcription regulation” in the type I interferon pathway. Specifically STAT3 protein interactions were determined using mass spectrometry analysis of proteins immunopurified by STAT3 pull down from HMC3 cell preparations.

### STAT3 Protein Complexes From Pull Down Studies

**Figure 5:**
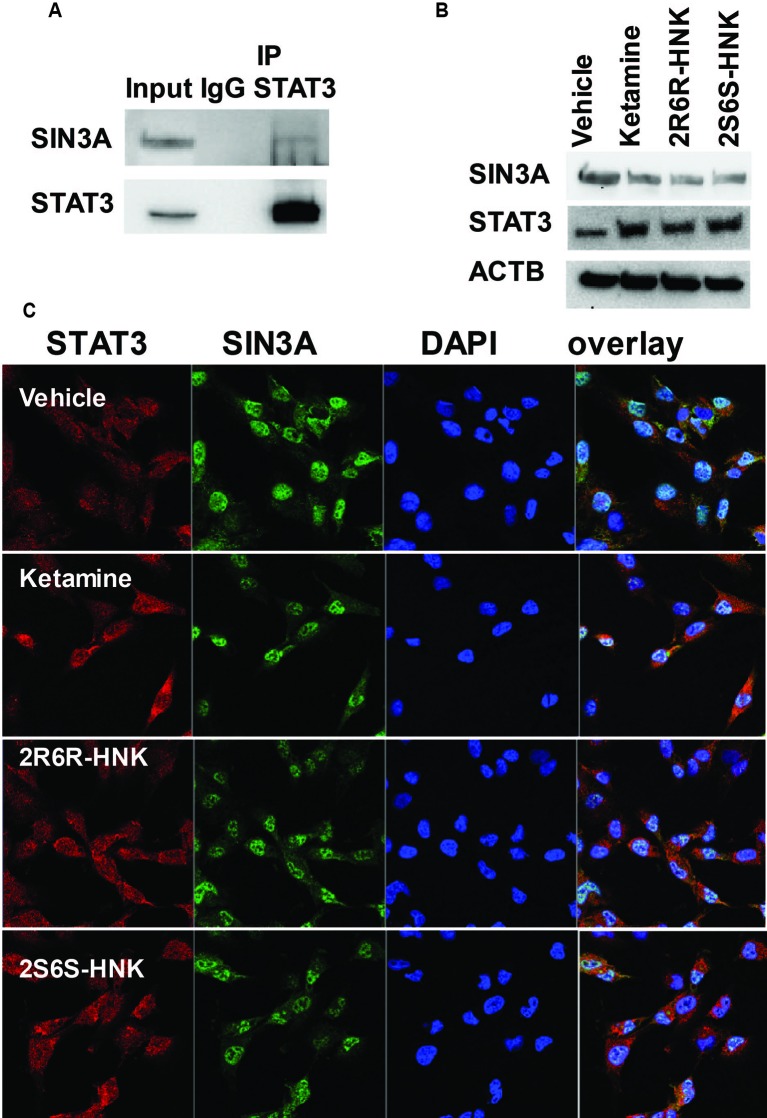
**(A)** Co-immunoprecipitation was used to determine whether STAT3 protein interacted with SIN3A in HMC3 cells. **(B)** STAT3 and SIN3A protein expression could be altered by exposure to ketamine, (*2R,6R*)-HNK or (*2S,6S*)-HNK. **(C)** Immunofluorescence staining of HMC3 cells showing the nuclear co-location of STAT3 and SIN3A after the cells had been treated with ketamine or its two active metabolites. Three biological replicates were performed.

We identified 721 proteins which could interact with STAT3 in HMC3 cells (**Supplementary Table 4**). We did not observe any proteins associated with response to the type I interferon pathway as shown in **Figure 3** among the STAT3 protein complexes determined by mass spectrometry. However, SIN3A, a transcription co-repressor of STAT3 is present in the STAT3 protein complex. We confirmed that observation by Co-IP (**Figure 5A**). It has been reported that the SIN3A repressor complex is a master regulator of STAT3 dependent transcriptional activity and is required for interferon stimulated gene transcription (Icardi et al., 2012). Specifically, knockdown of SIN3A substantially increased STAT3 expression in the nucleus after stimulation (Icardi et al., 2012). Our RNA-seq data showed that SIN3A expression was significantly downregulated in the presence of ketamine and its active two metabolites, while the expression of STAT3 was upregulated, results consistent with expression of these two proteins (**Figure 5B**). It should be pointed out that, in the presence of ketamine SIN3A was also co-localized with STAT3 in the nucleus (**Figure 5C**) where it acts as a transcription factor and can influence downstream gene expression.

**Figure 6:**
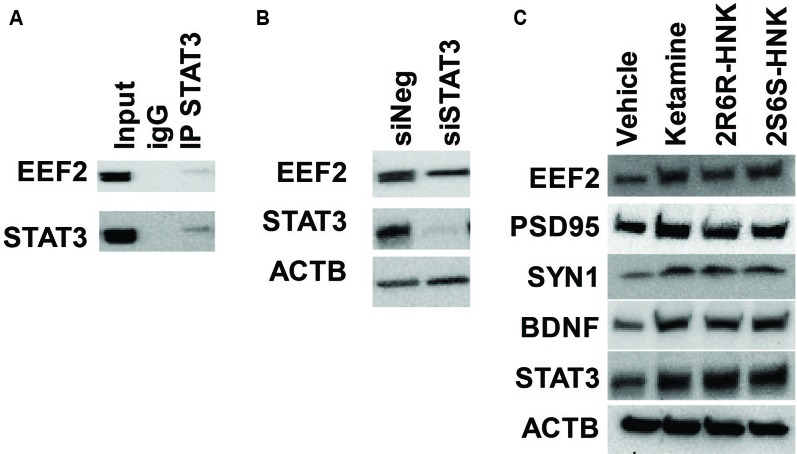
**(A)** Co-immunoprecipitation was used to determine whether STAT3 protein interacted with EEF2 in HMC3 cells. **(B)** Knockdown of STAT3 resulted in the down-regulation of EEF2 expression in HMC3 cells. **(C)** STAT3 could be induced by ketamine, (*2R,6R*)-HNK or (*2S,6S*)-HNK, and this effect was accompanied by augmentation of EEF2, PSD95, SYN1, and BDNF protein expression. Experiments were performed at least three times.

In addition, among the 721 genes in the STAT3 protein complex the mRNA expression for 570 genes (172 upregulated and 397 down-regulated) was significantly altered by ketamine (FDR < 0.05) based on our RNA-seq data. “Translation elongation” was the most significant pathway associated with genes included in the STAT3 protein complex. Furthermore, EEF2 protein was found to be the most abundant protein in the STAT3 protein complex. It was detected on the basis of 13 unique peptides corresponding to 21.2% amino acid sequence coverage spanning the EEF2 protein. We subsequently verified the interaction between STAT3 and EEF2 by Co-IP (**Figure 6A**). Moreover, knockdown of STAT3 resulted in the down-regulation in EEF2 in HMC3 cells (**Figure 6B**), and induction of STAT3 and of EEF2 was observed after cells were exposed to ketamine or its active metabolites (**Figure 6C**). This induction was accompanied by an upregulation of BDNF, PSD95 (also known as DLG4) and SYN1 after exposure to either ketamine or the two active ketamine metabolites as determined by RNA-seq and by Western blot analysis (**Figure 6C**), all protein that have been implicated as critical factors for synaptic plasticity. Furthermore, EEF2 can be regulated by EEF2 kinase which has been reported to play a role in the rapid antidepressant effects of ketamine (Monteggia et al., 2013). Ketamine treatment blocks NMDA receptor activity and leads to the inhibition of EEF2 kinase and the dephosphorylation of EEF2, which in turn promotes BDNF, PSD95 and SYN1 protein translation—all of which have been reported contribute to the antidepressant effects of ketamine (Zunszain et al., 2013).

In summary, this series of experiments suggests that an important transcription factor, STAT3, may play a significant role in response to ketamine therapy. That may be true, in part, as a result of STAT3 translocation into the nucleus in the presence of ketamine, which in turn, alters the expression of downstream genes involved in the type I interferon pathway. We also showed that STAT3 interacted with EEF2, a downstream target of mTOR signaling that is involved in the regulation of translation.

## Discussion

MDD is the most common psychiatric illness worldwide, with an estimated prevalence of approximately 7% in adults (Lépine and Briley, 2011). Esketamine has recently been approved by the Food and Drug Administration (FDA) for the treatment of “resistant depression,” defined as inadequate response to at least two antidepressant agents after adequate dosage and duration of therapy. Neuroinflammation plays an important role in the pathogenesis of depression and the response to antidepressants. Specifically, increased levels of pro-inflammatory cytokines, i.e., TNF, IL6, and IL1β, have been observed in depressed patients (Yang et al., 2015; Kiraly et al., 2017). It has also been shown that ketamine has anti-inflammatory properties that might contribute to its antidepressant effects (Yang et al., 2015), e.g., in patients with treatment resistant depression, serum IL6 concentrations were decreased only in patients who responded to ketamine therapy (Yang et al., 2015). However, it is unclear whether the active ketamine metabolites, i.e., (*2R,6R*)-HNK and (*2S,6S*)-HNK, also have anti-inflammatory properties.

The results of the present study indicate that STAT3 might not only play an important role in transcription regulation of genes involved in the type I interferon signaling pathway but also that it may play a role in protein translation regulation for synaptic plasticity markers. These results could help to increase our understanding of ketamine’s molecular and cellular mechanisms of action in microglia.

Our results have demonstrated that exposure to ketamine and two ketamine active metabolites results in similar gene expression profiles in microglia. Specifically, we found that “response to type I interferon” was the most significant pathway associated with changes in expression after treatment with ketamine and the two active ketamine metabolites that we studied (**Figure 3A**). They have also shown that changes in STAT3 intracellular localization (**Figure 2B**) by ketamine or its metabolites is associated with regulation of the expression of downstream genes (**Figures 4B–E**). STAT3 is an important transcription factor and signaling mediator for a series of proinflammatory cytokines such as IL6 and IL10 which can regulate important signaling pathways such as the NF-κB, AKT/PI3K/mTOR, and type I interferon pathways (van Boxel-Dezaire et al., 2006). These molecular mechanism(s) could be multifactorial in nature as a result of ketamine’s effects on neurotransmission, neuroinflammation and intracellular signaling. In addition, STAT3 might not only play an important role in immune response but also in the antidepressant effects of ketamine, effects that are mediated, at least in part, through interaction with EEF2. We demonstrated that STAT3 interacts with EEF2, and that both STAT3 and EEF2 can be induced by ketamine and its active metabolites (Figure 6C). EEF2 has been implicated in protein translation, synaptic plasticity and memory consolidation (Taha et al., 2013). It had been reported previously that ketamine could induce the dephosphorylation of EEF2, which in turn, resulted in increased expression of target genes such as BDNF which can trigger fast-acting antidepressant effects in mice (Monteggia et al., 2013). The role of STAT3 in both ketamine’s mechanism of action and in depression pathophysiology remains unclear. A recent preclinical study showed that ketamine activates JAK2/STAT3 signaling in the orbitofrontal cortex and improves stress-induced reversal learning deficit (Patton et al., 2017). Ketamine activates JAK2/STAT3 signaling through phosphorylation in cortical neurons, however, the effect is transient which might be due to negative feedback proteins such as SOCS3 (Patton et al., 2017). STAT3 also plays a major role in synaptic plasticity. Inhibition of STAT3 blocked NMDAR-long term depression, one of the major forms of synaptic plasticity in the brain (McGregor et al., 2017). Finally, a previous study showed that dysfunction of STAT3 in microglia might alleviate anti-depressant behavior in mice (Kwon et al., 2017).

Sex-dependent ketamine antidepressant effects have been observed in rodent models of depression (Carrier and Kabbaj, 2013; Franceschelli et al., 2015; Zanos et al., 2016; Sarkar and Kabbaj, 2016). However, a recent clinical trial suggested that there was not a significant difference between depressed men and women in ketamine response, and also that menopausal status did not influence response to ketamine therapy (Freeman et al., 2019). While many clinical and preclinical studies have provided evidence in support of ketamine’s rapid antidepressant effects (Scheuing et al., 2015; Zarate and Machado-Vieira, 2017), the mechanism of action of ketamine as an antidepressant remains unclear. Our pathway analysis of RNA-seq data demonstrated that gene expression profiles after E2 treatment differed from patterns seen after ketamine treatment (**Table 1**). However, in response to treatment with ketamine and treatment with ketamine plus E2, “response to type I interferon” was the pathway most significantly associated with changes in expression after drug exposure (**Table 1**). Although this pathway was in common between ketamine and ketamine plus E2 treatment, we found that the degree of change in expression levels appeared to differ (**Figure 1B**). Specifically, 155 genes could be up-regulated or down-regulated further (FDR < 0.05) in the presence of ketamine plus E2 as compared to ketamine or E2 treatment alone. Among these genes, the response to type I interferon pathway was once again observed during the pathway analysis (**Supplementary Table 3**).

The present study used a human microglial cell line, HMC3, to perform functional genomic studies. To our knowledge, this is currently the only commercially available human microglia cell line. Obviously, all cell lines have limitations. Therefore, our observations will have to be replicated by future studies conducted with clinical samples and/or patient-derived induced pluripotent stem cells (iPSCs) which can be differentiated into different types of central nervous system cells, i.e. iPSC-derived microglia. However, the results reported here represent a potentially important step in the process of obtaining functional insight into molecular mechanisms of action for ketamine and its active metabolites.

In summary, the present study has highlighted the fact that ketamine and its two active metabolites can regulate the type I interferon pathway mediated, at least in part, through STAT3. Specifically, genes that were modulated by either ketamine or its active metabolites were enriched in the “response to type I interferon” signaling pathway. Our data also raise the possibility that STAT3, which plays a critical role in the immune response, might also play a role in the antidepressant effects of ketamine, mediated in this case, at least in part, through EEF2, a protein that plays a role in BDNF, PSD95 and SYN1 translation—all of which are genes that have been shown to contribute to the antidepressant effects of ketamine.

## Data Availability Statement

The datasets generated for this study can be found in the NCBI, accession number GSE134782.

## Author Contributions

Participated in research design: M-FH, CZ, LZ, HL, and RW. Conducted experiments: M-FH and LZ. Performed data analysis and interpretation: M-FH, CZ, LZ, HL, and RW. Wrote or contributed to the writing of the manuscript: M-FH, LZ, and RW. All authors have given final approval of the version to be published.

## Funding

This work was supported in part by National Institutes of Health grants R01 GM28157, R01 AA27486, U19 GM61388 (the Pharmacogenomics Research Network), K01 AA28050, and by the Mayo Clinic Center for Individualized Medicine.

## Conflict of Interest

The authors declare that the research was conducted in the absence of any commercial or financial relationships that could be construed as a potential conflict of interest.

## Abbreviations

AMPA, α-amino-3-hydroxy-5-methyl-4-isoxazolepropionic acid; BDNF, brain-derived neurotropic factor; E2, estradiol; EEF2, eukaryotic elongation factor 2; ERα, estrogen receptor alpha; GSEA, gene set enrichment analysis; HNK, hydroxynorketamine; IP, immunoprecipitation; iPSC, induced pluripotent stem cells; MDD, major depressive disorder; NMDA, N-methyl-D-aspartate; PCA, principal component analysis; STAT, signal transducer and activation of transcription; TrkB, tyrosine kinase receptor B; IL6, interleukin 6; MCP-1, monocyte chemoattractant protein 1.
